# Sick Headache, Lobelia in

**Published:** 1840-07

**Authors:** 


### SICK-HEADACHE.

Dr. Burbell, of New York, in a letter to Dr. Alcott of Boston, says, "Not a case of sick-headache has ever occurred, within my knowledge, except with the drinkers of tea and coffee, and not a case had failed of being cured on the entire renunciation of them."

Is this the experience of physicians generally? If so, the fact ought to be known. The use of tea and coffee is almost universal, and it would probably be difficult to find a subject of sick-headache who was not in the habit of drinking them. But is it true, that entire abstinence from them will prevent or cure it? This is the important question, and if settled in the affirmative will go far to prove, that these drinks are the cause of sick-headache.

A lady of our acquaintance in Tennessee who had been for many years the victim of this malady, enjoys now a comparative exemption from it. Her attacks of the complaint were frequent and exceedingly severe. She ascribes her recovery to the use of the *tincture of Lobelia inflata*, which she took in doses of a teaspoonful, whenever she felt the premonitory symptoms of an attack. This quantity was just sufficient to excite nausea, but not to produce vomiting. The improvement in her health occurred about the 50th year of her age, and may have been the effect of some change of constitution at that period, rather than of the lobelia. The remedy, however, is worthy of trial in this most common and most troublesome affection. Y.

